# Biograft-HT^^®^^ as a bone graft material in the treatment of periodontal vertical defects and its clinical and radiological evaluation: Clinical study

**DOI:** 10.4103/0972-124X.60226

**Published:** 2009

**Authors:** K. T. Chandrashekar, Chhavi Saxena

**Affiliations:** *Department of Periodontics, Darshan Dental College, Udaipur, Rajasthan, India*; 1 *Department of Periodontics, Darshan Dental College, Udaipur, Rajasthan, India*

**Keywords:** Beta-triclacium phosphate, hydroxyapatite, vertical defects

## Abstract

**Aim:**

To determine the efficacy of Biograft-HT^®^ as a bone graft material in the treatment of vertical defects in generalized chronic periodontitis patients and their clinical and radiological evaluation.

**Patients and Methods:**

Twenty patients diagnosed with generalized chronic periodontitis having two or more vertical defects were selected for this study. Clinical parameters like plaque index, gingival index, probing pocket depth and clinical attachment levels were recorded at different points of time over six months. Radiographic evaluation included the depth of the bone defect and the percentage of bone defect fill and was carried out for both the groups at baseline, three months and six months. After recording clinical parameters and administering phase-1 therapy, the sites were randomly treated either with Biograft- HT^®^ or open flap debridement only.

**Results:**

At the end of six months there was a significant reduction in the plaque and gingival scores in both test and control groups. There was 64% decrease in probing pocket depth for the test site as compared to 54.52% decrease seen for the control group. Similarly there was an 84.82% gain in clinical attachment level from the baseline to six months post operatively for the experimental group in comparison to 68.83% gain for the control group. Furthermore, 43.57% bone fill was observed for the experimental site whereas only 17.98% of bone fill was evident in the control site.

**Conclusion:**

Biograft –HT improves healing outcomes, leads to a reduction of probing depth, a resolution of osseous defects and a gain in clinical attachment, compared with open flap debridement by itself.

## INTRODUCTION

Periodontal disease is one of the most prevalent diseases worldwide. Bacterial plaque has been implicated as the major etiological agent in the initiation and progression of inflammatory periodontal disease. Periodontal disease results in a loss of periodontal attachment apparatus, including loss of tooth-supporting alveolar bone. Although periodontitis is an infectious disease of the gingival tissue, changes that occur in the bone are crucial because the destruction of the bone is responsible for tooth loss.[1]

The purpose of periodontal therapy is to eliminate the inflammation of the periodontal tissues, to arrest the destruction of soft tissue and bone caused by periodontal disease, and regenerate the lost tissue, if possible.[2]

Bone grafting is the most common form of regenerative therapy and has been used for almost 100 years in attempts to stimulate healing of bony defects. The first recorded attempt to use a bone graft was by a Dutch surgeon, Job van Meckren, in 1668. However, the first use of a bone graft to rebuild bone loss by periodontal disease was reported by Hegedus in 1923. Materials such as plaster of paris, heterogenous bone powder, and other bone preparations were also tried for implantation into intrabony periodontal defects during the 1930s.[3]

Alloplastic bone graft materials are synthetic, inorganic, biocompatible, and bioactive bone substitutes that are believed to promote healing of bone defects through Osteoconduction.

Several calcium phosphate biomaterials have been tested since the mid-1970. They have excellent tissue compatibility and do not elicit any inflammation or foreign body response.[4,5] Two types of calcium phosphate ceramics have been used, Hydroxyapatite and tricalcium -phosphate.[6–8]

Biograft–HT^®^ (IFGL Bioceramics Limited, Calcutta) is a biphasic calcium phosphate consisting of hydroxyapatite and beta-tricalcium phosphate in the weight % ratio of approximately 70:30 that is biocompatible, non-toxic, resorbable, non-inflammatory, and bioactive. It causes no immunological, foreign-body, or irritating response, and has excellent oOsteoconductive ability.

The purpose of this clinical study was to evaluate this novel bone graft material, Biograft-HT^®^ in the treatment of intrabony defects and to evaluate it clinically as well as radiographically.

## PATIENTS AND METHODS

Twenty patients (with thirty defects) diagnosed with generalized chronic periodontitis having two or more vertical defects, were selected for this study from the Outpatient Department of Periodontics, Darshan Dental College, Udaipur (Rajasthan).

### Selection criteria

#### Inclusion criteria

1. Patients diagnosed as having generalized chronic periodontitis with probing depth of ≥5 mm and radiographic evidence of vertical bone loss
2. Age group of 35-55 years.
3. Patients with good general health, without any history of systemic disease

#### Exclusion criteria

1. Patients showing unacceptable oral hygiene during the presurgical (phase I) period
2. Pregnant women and lactating mothers
3. Smokers
4. Patients with systemically compromised status

### Study design

A written informed consent form explaining the nature of the study and surgical procedure was signed by the patient. Phase I therapy consisted of Oral Hygiene Instructions, scaling, root planning, and a prescription of chlorhexidine mouth wash. Patients were re-evaluated after phase-I therapy.

### Baseline recording of clinical parameters

Baseline measurements included Plaque Index, Gingival index, Probing pocket depth, and Clinical attachment level (using a UNC-15 probe with an occlusal stent).

### Radiographic parameters

An Intraoral periapical radiograph of each defect site was exposed using the long cone-paralleling technique. The mandibular molar region was the selected site for the study. Exposures were made at 70 KVP, 8 ma, 0.6 sec with inherent filtration of 2 mm AL. Kodak E-speed plus films. The film-to-object and focal spot-to-object distances were each standardized to 20 cms. Digitized images were displayed on the monitor at 5X magnification using Adobe Photoshop 7.0 computer software. A 0.5mm grid was made on the digitized images and all linear measurements were made using Auto-CAD 2006 software [Figures 1a and 2a].

**Figure 1a:**
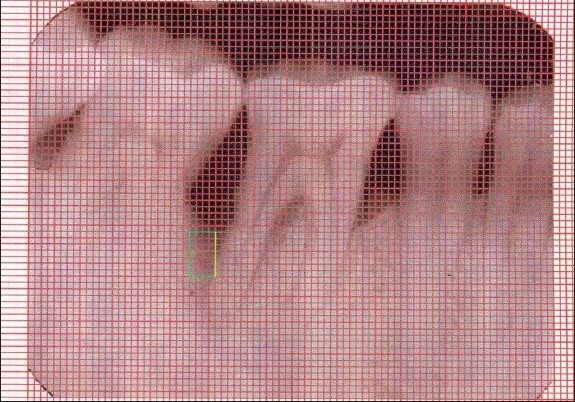
X-ray of control site at baseline

**Figure 1b:**
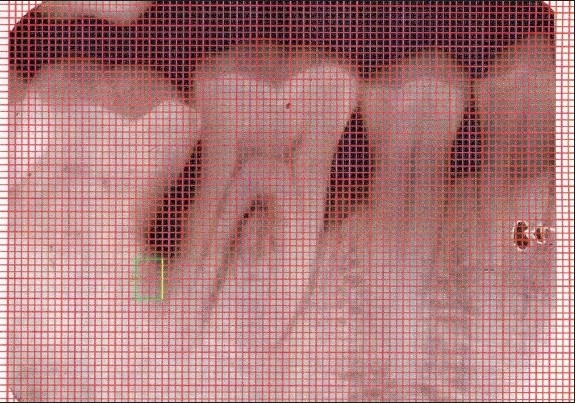
X-ray of control site after six months

**Figure 2a:**
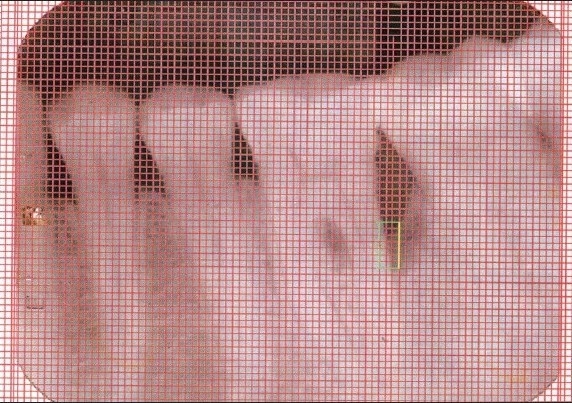
X-ray of experimental site at baseline

**Figure 2b:**
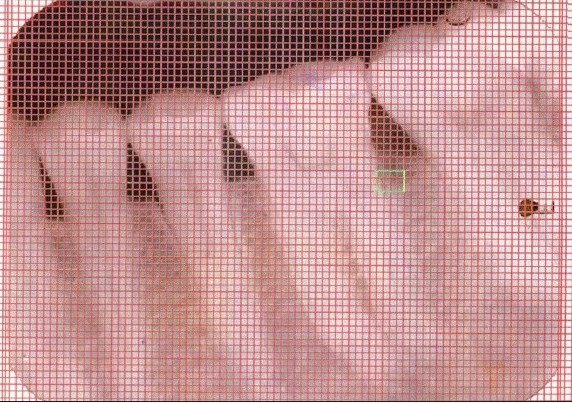
X-ray of experimental site after six months

### Pre-surgical protocol

Following an initial examination and treatment planning discussion, all the selected patients were given detailed instructions regarding the surgical procedure and then subjected to full mouth scaling, root planning and curettage with oral hygiene instructions. Occlusal adjustment was carried out wherever indicated; reevaluation was done after initial therapy.

All the patients were subjected to routine blood examination that included hemoglobin, bleeding time, clotting time, total leucocyte count, differential leucocyte count, and random blood sugar. An ELISA test was also carried out for HIV and Hepatitis screening.

All the sites were examined to record the clinical and radiographic parameters.

### Surgical protocol

The selected sites were randomly assigned to being either experimental or control sites. After adequate local anesthesia, crevicular incisions were made and the defect site was exposed by reflection of a full-thickness mucoperiosteal flap and debridement of the diseased granulation tissue, followed by thorough root planing and irrigation with normal saline [Figure 3a].

**Figure 3a:**
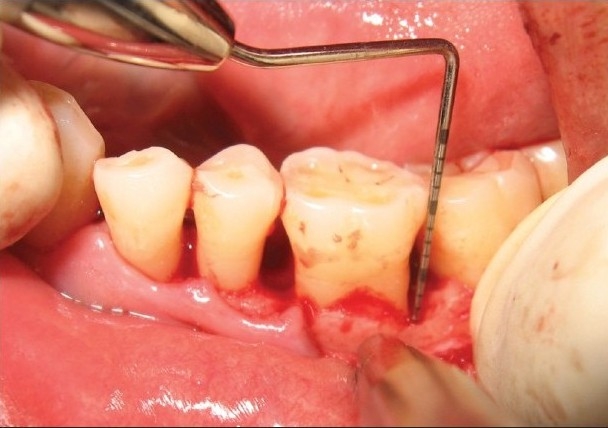
Experimental site showing vertical defect

At the experimental sites, the defect was filled with Biograft-HT^®^ (synthetic hydroxyapatite and beta-tricalcium phosphate) particles. The required quantity of graft material was transferred to a dappen dish, mixed with saline, and delivered into the osseous defect incrementally with the help of a Cumine Scaler (Hu-Friedy). The material was placed from the base of the defect coronally to the approximate level of the crest or the remaining osseous walls [Figure 3b]. The operative site was closed with 4-0 black silk sutures [Figure 3c] and protected with a noneugenol dressing.

**Figure 3b:**
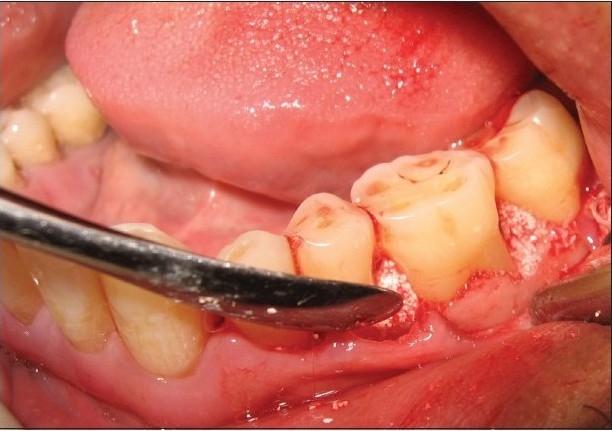
Experimental site with bone graft filled

**Figure 3c:**
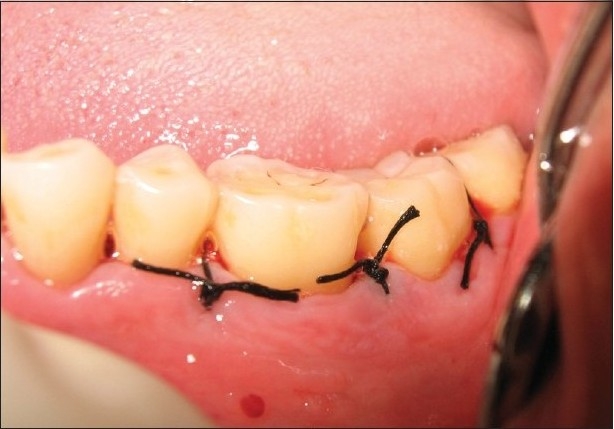
Experimental site after suturing

The control sites were left unfilled after surgical debridement, thorough root planning, and irrigation of surgical wound was done with normal saline [Figure 4a]. The mucoperiosteal flaps were repositioned and secured in place using black, braided (4-0), interrupted silk sutures to obtain primary closure of the interdental space [Figure 4b], and protected with a noneugenol dressing. All patients were prescribed an analgesic Diclofenac sodium 50 mg, twice a day, and Amoxycillin 500 mg thrice a day for five days.

**Figure 4a:**
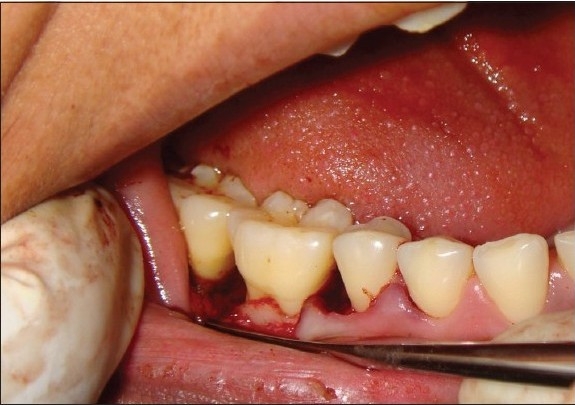
Control site showing defect after debridement

**Figure 4b:**
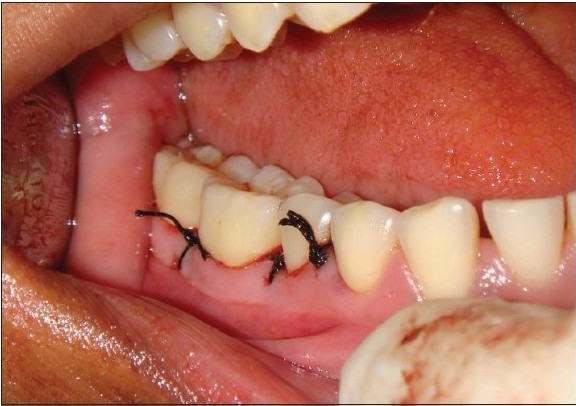
Control site after suturing

### Post-surgical protocol

After one week following surgery, the dressing and sutures were removed and the surgical site was irrigated thoroughly with saline. As healing was satisfactory and none of the patients experienced any untoward reaction, the recall appointments were made at one month, three, and six months. At each visit, oral hygiene instructions were reinforced and the surgical sites were professionally irrigated with normal saline.

At the end of three and six months post-therapy, patients were evaluated clinically and radiographically. Clinical parameters (plaque index, gingival index, probing pocket depth, clinical attachment level) and radiographic measurements were repeated for both control and experimental sites [Figures 1b and 2b].

## RESULTS

The primary goal of periodontal treatment is the maintenance of healthy and comfortable function of natural dentition. When periodontal disease results in a loss of the attachment apparatus, therapy aims at regeneration of the periodontal attachment, which includes the formation of new cementum, functionally oriented periodontal ligament and alveolar bone on the root surface.

The present clinical study was aimed at evaluating the effectiveness of Biograft-HT^®^ as bone grafting material in the treatment of vertical defects in generalized chronic periodontitis patients, and to compare its effectiveness to open flap debridement by itself. Biograft-HT^®^ is a new bone graft material consisting of hydroxyapatite and β-tricalcium phosphate in the ratio of 70:30. The particle size of the graft material was 250 μ.

### Plaque index

No statistically significant differences were found in the mean values for the plaque index between the test and control groups at baseline (*P* = 0.173), one month (*P* = 0.956), three months (*P* = 0.729), and six months (*P* = 0.181) [Table 1].

**Table 1:** Mean plaque index before and after treatment

Plaque Index	Control group		Experimental group		*P* value
			
	Mean ± SD	% Change from baseline (%)	Mean ± SD	% Change from baseline (%)
Baseline	1.465 ± 0.128	-	1.397 ± 0.136	-	0.173
1 month	1.203 ± 0.198	17.88	1.207 ± 0.193	13.60	0.956
3 months	0.844 ± 0.196	42.38	0.819 ± 0.190	41.37	0.729
6 months	0.655 ± 0.174	55.29	0.575 ± 0.144	58.84	0.181
*P* value	<0.000**		<0.000**		

### Gingival index

No statistically significant differences were found in the mean values for the gingival index between the test and control groups at baseline (*P* = 0.069), at one month (*P* = 0.050), three months (*P* = 0.060), and six months (*P* = 0.172) [Table 2].

**Table 2:** Mean gingival index before and after treatment

Plaque index	Control group		Experimental group		*P* value
			
	Mean ± SD	% Change from baseline	Mean ± SD	% Change from baseline	
Baseline	1.09 ± 0.2134	-	0.9407 ± 0.2190	-	0.069
1 month	0.9013 ± 0.1932	17.31	0.7627 ± 0.1779	18.92	0.050*
3 months	0.6973 ± 0.1102	36.02	0.6173 ± 0.1129	34.37	0.060
6 months	0.5220 ± 0.114	52.11	0.4713 ± 0.0850	49.89	0.172
*P* value	<0.000**		<0.000**		

### Probing pocket depth

**Graph 1:**
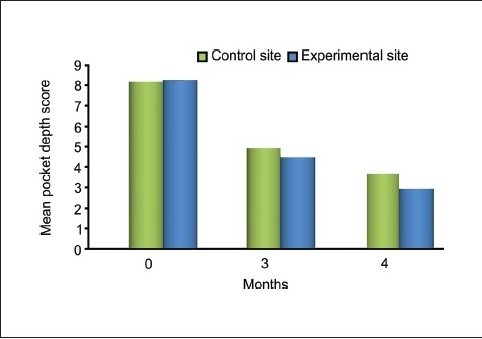
Comparing pocket depth score between experimental and control site mean clinical

No statistically significant differences were found between the test and control groups at baseline (*P* = 0.646) and three months (*P* = 0.109). However, the mean values at six months (*P* = 0.014) were highly significant. The decrease in probing depth in the experimental site from baseline to six months postoperation was 64.26% as compared to the control group which showed a decrease of 54.52% [Table 3 and Graph 1].

**Table 3:** Mean probing depth before and after treatment

Probing pocket depth	Control group		Experimental group		*P* value
			
	Mean ± SD	% Change from baseline	Mean ± SD	% Change from baseline	
Baseline	8.07 ± 0.88	-	8.20 ± 0.68	-	0.646
3 months	4.93 ± 0.70	38.90	4.47 ± 0.83	45.48	0.109
6 months	3.67 ± 0.72	54.52	2.93 ± 0.80	64.26	0.014*
*P* value (baseline-3 months)	0.000**		0.000**		
*P* value (baseline-6 months)	0.000**		0.000**		

### Clinical attachment level

**Graph 2:**
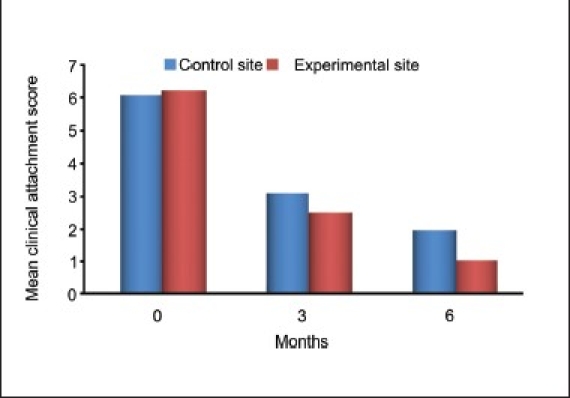
Mean clinical attachment lever score between experimental and control site

**Graph 3:**
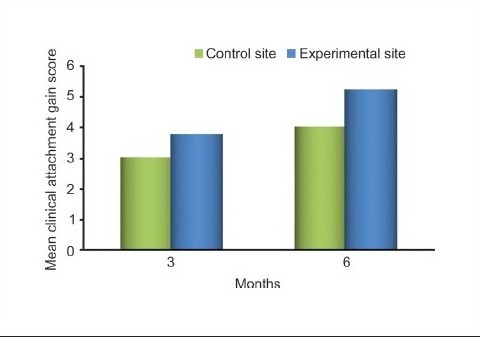
Comparing clinical attachment gain score between experimental and control site

The difference between the mean values for the levels of clinical attachment at baseline (*P* = 0.65) in the test and control groups was not significant. However, the differences in the mean values of clinical attachment levels at three (*P* = 0.036) and six months (*P* < 0.001) were statistically significant. This gain in clinical attachment from the baseline to six months postoperatively was 84.82% for the experimental group and 68.83% for the control group [Table 4 and Graphs 2 and 3].

**Table 4:** Mean clinical attachment level before and after treatment

Clinical attachment level	Control group		Experimental group		*P* value
			
	Mean ± SD	% Change from baseline	Mean ± SD	% Change from baseline	
Baseline	6.00 ± 0.85		6.13 ± 0.74	-	0.65
3 months	3.00 ± 0.65	50	2.40 ± 0.83	60.84	0.036*
6 months	1.87 ± 0.64	68.83	0.93 ± 0.80	84.82	0.001**
*P* value (baseline-3 months)	<0.000**		<0.000**		
*P* value (baseline-6 months)	<0.000**		<0.000**		

* *P*<0.01- significant

** *P*<0.001- highly significant

### Amount of bone fill in the defects

**Graph 4:**
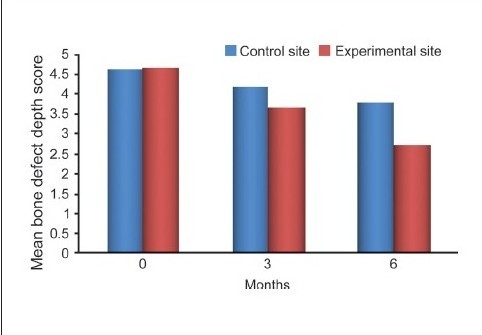
Comparing bone defect depth score between experimental and control site

For control sites, the statistically significant mean difference in defect fill from the baseline was 4.2000 ± 0.9783 mm (*P* = 0.212) at three months and 3.8000 ± 0.8619 mm (*P* = 0.014) at six months. For experimental sites, the statistically significant mean difference in defect fill from baseline was 3.6667 ± 1.0293 mm (*P* = 0.013) at three months and 2.6333 ± 0.8958 mm (*P* < 0.001) at six months [Table 5 and Graph 4].

**Table 5:** Mean bone fill of defect before and after treatment

Depth of the defect	Control group		Experimental group		*P* value
			
	Mean ± SD	% Change from baseline	Mean ± SD	% Change from baseline	
Baseline	4.6333 ± 0.8756	-	4.6667 ± 1.0465	-	0.925
3 months	4.2000 ± 0.9783	09.35	3.6667 ± 1.0293	21.42	0.157
6 months	3.8000 ± 0.8619	17.98	2.6333 ± 0.8958	43.57	0.001**
*P* value (baseline to 3 months)	<0.212		<0.013*		
*P* value (baseline to 6 months)	<0.014*		<0.000**		

* *P*<0.01- significant

** *P*<0.001- highly significant

**Graph 5:**
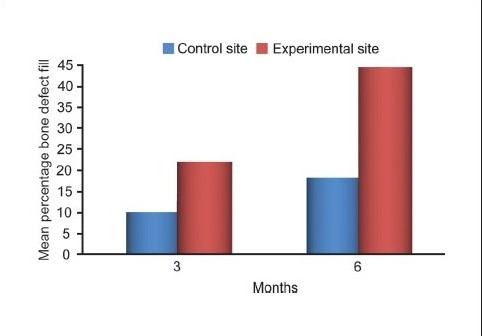
Comparing bone defect percentage fill between experimental and control site

The differences in the mean values of the amount of defect fill at baseline (*P* = 0.925) and at three months (*P* = 0.157) were not significant but the difference was statistically significant at six months (*P* < 0.001) between the experimental and control groups [Graph 5].

## DISCUSSION

The primary goal of periodontal treatment is the maintenance of healthy and comfortable function of natural dentition. When periodontal disease results in a loss of the attachment apparatus, therapy aims at regeneration of periodontal attachment that includes formation of new cementum, functionally oriented periodontal ligament, and alveolar bone on the root surface.

Various modalities of regeneration available these days include the root surface biomodification, guided tissue regeneration techniques, and bone grafts. Guided tissue regeneration is based on the principle of guiding the proliferation of various periodontal tissue components during healing following periodontal surgery to achieve regeneration. Placement of a barrier membrane (resorbable or nonresorbable) between the soft tissue flap and root surface favored the repopulation of the wound area adjacent to the root by regenerative cells originating from the periodontal ligament. Biological mediators to enhance cellular repopulation of the periodontal wound are also available, including peptide sequences, protein preparations, and growth factors to regenerate tissues through the principle of mimicking the natural process of tooth formation with the expectation that the regeneration cascade will proceed spontaneously. Such peptides include emdogain, platelet-rich plasma preparation etc. Bone grafts and bone substitutes used in regenerative therapy are derived from bone or non-osseous materials, and correction of the loss of alveolar bone occurs by osteoconduction phenomenon.

A synthetic bone substitute has been used in this study and clinical parameters like plaque index, gingival index, probing pocket depth, and clinical attachment level were compared. An attempt was also made to compare the results radiographically. Clinical parameters were recorded at the baseline, three months, and six months after the operation. The present study was a six months' follow-up study based on the concept that dimensional alterations of the periodontal tissues resulting from active therapy occur within the first six months. The most reliable outcome for assessing periodontal regeneration is human histological investigation, but this is precluded by practical and ethical constraints due to the associated morbidity.

Comparative analysis of plaque index scores of the control and experimental sites at baseline revealed mean scores of 1.465 ± 0.128 and 1.397 ± 0.136 respectively. At the end of six months, the mean value of the control site decreased to 0.655 ± 0.174 whereas the experimental site decreased to 0.575 ± 0.144. This resulted in a ‘t’ value of 1.371 (*P* < 0.181), indicating a non-significant difference between the two sites. However, the, change in plaque index scores from baseline to six months was significant for both the experimental and control sites, which could be attributed to the rigorous oral hygiene maintenance regime, regular follow-up visits, and reinforcement of oral hygiene instructions for the patients throughout the study period. These results are comparable to previous studies reported by Oreamuno *et al*.[9]

Comparative analysis of gingival index scores of the control and experimental sites at baseline revealed mean scores of 1.0900 ± 0.2134 and 0.9407 ± 0.2190 respectively (*P* < 0.069), indicating a no-significant difference between the two sites. At the end of six months, the mean value of the control site decreased to 0.5220 ± 0.1114 and whereas that of the experimental site decreased to 0.4713 ± 0.1004 (*P* < 0.172 between the two sites). This improvement in gingival status could be due to the surgery and frequent supportive therapy provided. Similar findings were reported by Yukna *et al*.[10]

Comparative analysis of the control and experimental sites at baseline revealed probing pocket depth scores of 8.07 ± 0.88 and 8.20 ± 0.68 respectively (*P* < 0.646), indicating a non-significant difference between the two sites. At three months postoperatively, the values showed a mean of 4.93 ± 0.70 for the control site and 4.47 ± 0.83 for the experimental site, indicating a slightly significant difference (*P* < 0.109). At the end of six months, the mean value of the control site decreased to 3.67 ± 0.72 whereas that for the experimental site decreased to 2.93 ± 0.80, resulting in a highly significant difference (*P* < 0.014) between the two sites. This decrease in probing depth in the control group was less than that of the experimental group, which was also statistically significant. This compares favorably with the studies done earlier by Yukna *et al.*, Kreji *et al.*, Nery *et al.*, Stahl and Forum, and Galgut.[4,11–14]

Comparative analysis of the control and experimental sites at baseline revealed clinical attachment level scores of 6.00 ± 0.85 and 6.13 ± 0.74 respectively (*P* < 0.650). At three months postoperatively, the values showed means of 3.00 ± 0.65 for the control site and 2.40 ± 0.83 for the experimental site, indicating a significant difference (*P* < 0.036) At the end of six months, the control site's clinical attachment level had decreased to 1.87 0.64 whereas the value for the experimental site decreased to 0.93 ± 0.80, indicating a statistically highly significant difference (*P* < 0.001). The comparable gain in the clinical attachment level of control group could be attributed to the formation of the long junctional epithelium instead of increased bone fill and tissue repair as seen in the experimental group. However, the nature of this attachment could not be elicited as it required histological evaluation. This finding is consistent with those of the studies reported by Nery *et al.*, Galgut, Bowen *et al.*, and Reynolds.[13–16]

The depth of the defect was the distance from the alveolar crest to the base of the defect. Comparative analysis of the mean percentage change in defect fill for both the sites revealed a about 43.57% bone fill for the experimental site and 17.98% for the control site. Thus, the experimental site had a higher percentage of defect fill than did the control site, the difference being statistically highly significant. These findings are consistent with those of Nery *et al.*, Stahl and Forum, Galgut, and Meffert *et al*.[4,13,14,17,18]

## CONCLUSIONS

This new combination of synthetic hydroxyapatite and β-tricalcium phosphate (Biograft-HT^®^) proved to be biocompatible and showed improved healing outcomes as compared with open-flap debridement by itself. These outcomes included a reduction of probing depth and a gain in clinical attachment level and amount of bone fill in the defects. There was a significant reduction in the probing pocket depth and a gain in clinical attachment level in both the experimental and control sites. However, the site implanted with the graft material showed a higher reduction in pocket depth and a higher gain in clinical attachment level compared to the control site. Radiographic assessment showed greater defect fill in the experimental site as compared to the control group, indicating the efficacy of the graft material.

Although Biograft-HT^®^ has shown promising results on clinical and radiographic evaluation, additional long-term studies should be undertaken to obtain more clinical evidence for regular use of this material.
